# Assessing Distinctiveness in Multidimensional Instruments Without Access to Raw Data – A Manifest Fornell-Larcker Criterion

**DOI:** 10.3389/fpsyg.2020.00223

**Published:** 2020-03-09

**Authors:** Frederic Hilkenmeier, Carla Bohndick, Thomas Bohndick, Johanna Hilkenmeier

**Affiliations:** ^1^Psychology School, Fresenius University of Applied Sciences, Hamburg, Germany; ^2^Universität Hamburg, Hamburg, Germany; ^3^Independent Researcher, Hamburg, Germany

**Keywords:** validity evidence based on internal structure, Fornell–Larcker criterion, reliability, distinctiveness, multidimensional measurement instruments

## Abstract

The assessment of an instrument’s conceptual framework as prerequisite for conducting further analyses has been advocated for decades. Multidimensional instruments posit several components that are each expected to be homogeneous but distinct from each other. However, validity evidence supporting the proposed internal structure is often missing. This leaves researchers and practitioners who are interested in a certain instrument in a precarious situation: Before starting their own data collection, they do not know whether dimensions adequately discriminate from each other and thus whether they can have confidence in any interpretation of these dimensions. Adapting the Fornell–Larcker criterion, we propose estimating distinctiveness between dimensions by using nothing but the most commonly reported statistics: Cronbach’s alpha and the correlation matrix between the manifest composite scores of the dimensions in question. A simulation study demonstrates the usefulness of this “manifest Fornell–Larcker criterion” in providing an easily assessable method for vetting existing instruments, whereas a systematic literature review shows the necessity to do so even for instruments published in well-received journals.

## Introduction

In psychology, we are often interested in concepts that cannot be observed directly. For instance, social psychology deals with “attitudes” or “intention,” work and organizational psychology is often interested in “motivation” or “commitment,” and educational psychology tries to figure out the effect of “teacher expectancy” on “verbal ability,” to name just a few. These concepts are theoretical in nature. They provide a degree of abstraction that permits us to generalize relationships (Bollen, 2002). Since they cannot be observed directly, those variables cannot be assessed directly either (Jöreskog and Sörbom, 1979). Instead researchers “must operationally define the latent variable of interest in terms of behavior believed to represent it. As such, the unobserved variable is linked to one that is observable, thereby making its measurement possible” (Byrne, 1998, p. 4). These observable manifest variables serve as indicators of the underlying latent ones that they are presumed to represent. Identifying and selecting fitting indicators is thus crucial for the assessment of the underlying latent variable (Byrne, 1998). It is even more crucial since psychology is a cumulative science in which new research builds on existing one, replicates it, and extends it (Open Science Collaboration, 2015). Therefore, it is necessary to use “common tools” to obtain robust, replicable, and consequential findings (Mischel, 2009).

How to develop such “common tools” in the form of measurement instruments that allow for consequential decisions about individuals or systems has been the topic of methodological papers and standards for decades, for example, in the “Standards for Educational and Psychological Testing” (American Educational Research Association [AERA] et al., 1999, 2014).

Following Messick’s (1995) influential work, the standards identify validity as most fundamental in developing and evaluating measurement instruments (American Educational Research Association [AERA] et al., 2014). Ideally, critical validity evidence can be retrieved from the paper introducing the instrument. However, as Brennan (2006) stated, “validity theory is rich, but the practice of validation is often impoverished”, p. 8). Indeed previous reviews of the literature indicate that most studies severely lack these vital information (e.g. Cizek et al., 2008; Zumbo and Chan, 2014). Following the procedure of an ongoing validational process outlined in the standards, the present study demonstrates a method by which one of the sources of validity evidence, namely evidence based on internal structure, can be determined for already published multidimensional instruments without access to raw data, i.e. from information “available from earlier reported research” (American Educational Research Association [AERA] et al., 2014, p. 21).

This article has five parts. First, we present a brief overview over evidence based on internal structure and its current reporting practice. Second, we compare different methods to assess the internal structure, more specifically the distinctiveness in multidimensional measurement instruments. Third, we propose how one of these methods, namely the Fornell–Larcker criterion (Fornell and Larcker, 1981), can be estimated for already published instruments by using nothing but the two most commonly reported statistics: Cronbach’s alpha and the correlation matrix between the manifest composite scores of the components in question. Fourth, we demonstrate that this “manifest Fornell–Larcker criterion” can be used to discern lack of distinctiveness in multidimensional instruments by means of a simulation study. Finally, we test its real-life usefulness for already published instruments by means of a systematic review.

## Evidence Based on Internal Structure

A prerequisite for the validational process is a detailed specification of the conceptual framework the instrument is intended to measure. Multidimensional instruments posit several components that are each expected to be homogeneous but also distinct from each other. Evidence based on internal structure reflects the degree to which the relationships among items and components conform to this conceptual framework (American Educational Research Association [AERA] et al., 2014).

As noted by Campbell and Fiske as early as 1959 (Campbell and Fiske’s, 1959, p. 84): “One cannot define without implying distinctions, and the verification of these distinctions is an important part of the validational process.” Therefore, testing whether a component does “not correlate too highly with measures from which it is supposed to differ” (Campbell, 1960, p. 84) “must be prior to the testing of other propositions to prevent the acceptance of erroneous conclusions” (Campbell and Fiske’s, 1959, p. 100). Distinctiveness between components of a multidimensional instrument (also referred to as “subscales,” “dimensions,” or “facets”) is needed to demonstrate not only conceptual but also empirical distinctness among them (Shiu et al., 2011).^1^ Otherwise, the components are not unique but capture phenomena also represented by other components, making any interpretation of differences between them likely a result of statistical discrepancies (Henseler et al., 2015). Likewise, due to multicollinearity between the components, any conclusions made regarding relations to other variables may be incorrect as well (Farrell, 2010). In short, distinctiveness has to be ensured during the instrument development process. Otherwise, the interpretation of the instrument itself, the relations to other variables, and thus the interpretation of any resulting scores are highly questionable (Block, 1963; Fornell and Larcker, 1981; Messick, 1995; Farrell, 2010; Schmidt, 2010, Hair et al., 2014).

Therefore, the assessment and the establishment of the internal structure of a measurement instrument, and especially the distinctiveness of components of multidimensional scales, is not only one of the most important but also one of the most overlooked sources of validity evidence. At first glance, this statement might be surprising given that systematic reviews about current validational practice like that of Cook et al. (2014) show that most papers already include information about internal structure. Yet a closer inspection shows that the overwhelming majority of the studies included in the systematic reviews view internal structure as reliability evidence and only report values like Cronbach’s alpha instead of viewing internal structure as validity evidence and actually testing the proposed structure of the conceptual framework the instrument is supposed to measure. Crutzen and Peters (2017) showed that about 71% off all multidimensional instruments in their systematic review reported Cronbach’s alpha, but only about 16% actually assessed dimensionality, i.e. 84% of the multidimensional instruments under investigation did not report any validity evidence regarding their internal structure at all. Likewise, Cizek et al. (2008) report that only 8.5% of the 283 instruments they investigated viewed internal structure as bearing on validity [also see Cook et al. (2014)]. This is diametrical to the *Standards*, which states that “such an index [like Cronbach’s alpha] would be inappropriate for tests with a more complex internal structure” (American Educational Research Association [AERA] et al., 2014, p. 16; also see Standard 1.13 and Standard 1.14).

## Assessment of Distinctiveness in Multidimensional Measurement Instruments

Besides the general recommendations for explorative and confirmatory factor analyses [which are usually reported insufficiently, see e.g. Schmitt and Sass (2011)], the literature reveals several ways for specifically analyzing the distinctiveness in multidimensional instruments. All of these recommendations rely on assessing the intercorrelation between the posited dimensions and a cutoff criterion to determine whether distinctiveness between dimensions is met. For instance, Brown (2006) suggests that dimensions with correlation exceeding 0.80 or 0.85 should be collapsed into a single factor since the notion that they represent distinct components is untenable (p. 131; p. 158). Likewise, Kline (2010) states that at a correlation of 0.90, “we can hardly say that variables *X* and *Y* measure distinct [components]” (p. 71). In a similar vein, Bagozzi et al. (1991) argue that distinctiveness between two dimensions is achieved when the correlation between them significantly differs from 1.0. This approach is conceptually identical to the popular CFA model comparison of a one-factor solution to a two-factor solution via a chi-square difference test (Brown, 2006, p. 163).

However, we would argue that the aforementioned approaches are not without caveats: For one, the CFA model comparison and the test against a correlation of 1.0 are extremely liberal; they only test whether two dimensions are not measuring exactly the same, which is a pretty high bar given the measurement error inherent in psychological instruments. As Anderson and Gerbing (1988) put it: “Although this is a necessary condition for demonstrating discriminant validity, the practical significance of this difference will depend on the research setting.” The rule-of-thumb criteria of 0.80, 0.85, or 0.90 (see Brown, 2006 and Kline, 2010), on the other hand, are too inelastic to account for the fidelity bandwidth dilemma: Two related components can correlate quite high but still measure something distinctly different as long as they are of a narrow bandwidth (i.e. consist of more homogenous items). Contrariwise, two components of a broader bandwidth (i.e. consisting of more heterogeneous items), which are by nature more abstract and inclusive (Ones and Viswesvaran, 1996), have to differ more strongly from each other to be differentiable and each measures something unique.

To circumvent these caveats, Fornell and Larcker (1981) propose the comparison of two measures of variance: First, a variable’s average variance is extracted (AVE), which represents the average amount of variance that a variable explains in its indicators and, second, the squared intercorrelations between the variables in the contextual framework, representing the amount of variance a variable shares with each other variable. Distinctiveness is established when a variable is more closely related to its own indicators than to those of any other variable within the contextual framework.

The formal definition of the AVE of a given latent variable *X* with standardized indicators can be seen in Equation (1).

(1) $$A\,V\,E_{x}=\frac{\sum \lambda_{x.i}^{2}}{K_{x}}$$

where $\lambda_{x.i}^{2}$ is the squared loading of indicator_*x.i*_ on the latent variable *X*, and *K*_*x*_ is the number of indicators associated with *X*. As can be seen in Equation (2), the Fornell–Larcker criterion and thus the requirements for distinctiveness between two latent variables *X* and *Y* are fully met if the AVE of *X* and *Y* are both higher than the variance that *X* and *Y* share with each other.

(2) $$A\,V\,E_{x}>\varphi_{x\,y}^{2}\,\text{and}\,A\,V\,E_{y}>\varphi_{x\,y}^{2}$$

where $\varphi_{\text{xy}}^{2}$ is the squared correlation between *X* and *Y*.

The underlying idea is similar to the one expressed in Campbell and Fiske’s 1959, p. 83) interpretation of the multitrait–multimethod matrix (MTMM), stating that the reliability of a component should be higher than its correlation “with measures designed to get at different traits.” Thus, as the MTMM, the Fornell–Larcker criterion is neither conservative nor liberal *per se* when determining distinctiveness. The correlative threshold ($\varphi_{\text{xy}}^{2}$) varies with the reliability of the measure. Compared to the (latent) MTMM, the main advantage of the Fornell–Larcker criterion is its parsimony. The MTMM requires that each concept in question is measured by at least two different methods (e.g. self-reports and peer ratings), which is seldom the case in psychological research (Achenbach et al., 2005). Thus, the Fornell–Larcker criterion can be used more widely than the MTMM since it only requires a single measurement method, and indeed in other disciplines of social sciences, it is the most commonly used way to assess distinctiveness (e.g. Shiu et al., 2011). However, it is severely underutilized in psychological research, even though it can easily be calculated from any statistical package designed for structural equation modeling.

To summarize, only a fraction of studies using multidimensional instruments actually report on the dimensionality of the instrument at all (Crutzen and Peters, 2017), and those who do often report EFA or CFA results in a way that is insufficient for determining the internal structure of multidimensional instruments (Schmitt and Sass, 2011). There are more specific methods to assess distinctiveness between dimensions. However, these methods are either extremely liberal (Bagozzi et al., 1991), inelastic rules-of-thumb (Brown, 2006; Kline, 2010), requiring a much more complex data collection and are therefore seldom used (Campbell and Fiske’s, 1959), or are underutilized in psychological research despite the availability in statistical packages (Fornell and Larcker, 1981). Overall, it is more likely than not that articles do not contain information on whether dimensions adequately discriminate from each other and thus whether one can have confidence in any interpretation of these dimensions (Crutzen and Peters, 2017).

## A Manifest Fornell–Larcker Criterion

To make the best out of this precarious situation and help researchers and practitioners *a priori* estimate the distinctiveness of the dimensions of a given instrument, we propose using Cronbach’s alpha as an adequate substitute for AVE and the correlation matrix between the composite scores as an adequate substitute for $\varphi_{x\,y}^{2}$. The mathematical derivations of these substitutes are nothing new and well documented in methodological papers and textbooks for decades. What we newly suggest is using these substitutes to calculate a manifest Fornell–Larcker criterion. This manifest Fornell–Larcker criterion is an *auxiliary tool* for cases in which the article introducing a given multi-dimensional instrument neither addresses distinctiveness directly nor provides the necessary information to compute the original Fornell–Larcker criterion (i.e. all factor loadings and latent correlations between the subdimensions). Given the results of previous systematic reviews on reporting validity based on the internal structure (Cizek et al., 2008; Cook et al., 2014; Crutzen and Peters, 2017), such an *auxiliary tool* is needed more often than not.

To establish the general principle of our procedure, we will assume essentially tau-equivalent data. A more detailed derivation of the equations used as well as an adaptation to the congeneric model, which is much more realistic for empirical data but at the same time fuzzier when it comes to deriving the equations, can be found as Electronic Supplementary Material. As shown in Equation 1, AVE represents the average amount of variance that a variable explains in its indicators and therewith can be interpreted as a measure of reliability. As evidenced by McNeish (2018), no other statistic is reported more often as an indicator of a test score’s reliability than Cronbach’s alpha, and indeed if the items of an instrument are essentially tau-equivalent, Cronbach’s alpha is a true indicator of that instrument’s reliability (Raykov, 1997). The formula for standardized Cronbach’s alpha is as follows:

(3) $$\alpha_{x}=\frac{\sum r_{i}}{1+\left(K_{x}-1\right)*\left(\frac{\sum r_{i}}{K_{x}}\right)}$$

where α_x_is the standardized Cronbach’s alpha of all indicators associated with the latent variable *X*, *K*_x_ is the number of indicators associated with *X*, and *r*_i_ is the inter-item correlation of indicator *i* with all other indicators associated with *X*. Given Equation (3), in the essentially tau-equivalent model, Cronbach’s alpha and the number of items are sufficient to calculate the AVE.

(4) $$A\,V\,E_{x}=\frac{\sum \lambda_{x.i}^{2}}{K_{x}}={\left(\frac{\sum \lambda_{x.i}}{K_{x}}\right)}^{2}=\frac{\sum r_{i}}{K_{x}}=\frac{\alpha_{x}}{\alpha_{x}*\left(-K_{x}\right)+\alpha_{x}+K_{x}}$$

Again, in the Fornell–Larcker criterion, the variance a latent variable *X* shares with its indicators (*AVE*_x_) is counterbalanced by the variance it shares with any other latent variable within the conceptual framework ($\varphi_{\text{xy}}^{2}$). However, many papers only report the correlation matrix between the composite scores (i.e. the summed scores or mean scores), not between the latent ones. Unlike correlations between latent variables, correlations between manifest variables do not take measurement error into account. One can “correct” for this attenuation by utilizing the reliability of the variables (e.g. Block, 1963). Since in the essentially tau-equivalent model Cronbach’s alpha is a true measure of reliability, the latent correlation can be substituted as shown in Equation (5),

(5) $$\varphi_{x\,y}=\hat{r_{x\,y}}=\frac{r_{x\,y}}{\sqrt{\alpha_{x}}*\sqrt{\alpha_{y}}}$$

where $\hat{r_{\text{xy}}}$ is the “corrected” (“double-corrected”, to be more precise) correlation between the two composite scores of *X* and *Y*, and *r*_xy_ is the manifest correlation between the two composite scores of *X* and *Y*. This shows that in the essentially tau-equivalent model, distinctiveness can indeed be calculated using nothing but Cronbach’s alpha and the manifest correlation between the composite scores.

$A\,V\,E_{X}>\varphi_{x\,y}^{2}\,\mathrm{which}\,\mathrm{is}\,\mathrm{equivalent}\,\mathrm{to}$

$$\frac{\alpha_{x}}{\alpha_{x}*\left(-K_{x}\right)+\alpha_{x}+K_{x}}>\frac{r_{x\,y}}{\sqrt{\alpha_{x}}*\sqrt{\alpha_{y}}}\,\text{and}$$

$A\,V\,E_{y}>\varphi_{x\,y}^{2}\,\mathrm{which}\,\mathrm{is}\,\mathrm{equivalent}\,\mathrm{to}$

(6) $$\frac{\alpha_{y}}{\alpha_{y}*\left(-K_{y}\right)+\alpha_{y}+K_{y}}>\frac{r_{x\,y}}{\sqrt{\alpha_{x}}*\sqrt{\alpha_{y}}}$$

The derivation for the congeneric model can be found in Electronic Supplementary Material. However, what is important is that, in the congeneric model, Cronbach’s alpha will underestimate the reliability of the measurement instrument (Raykov, 1997); thus, the approximation of the AVE shown in Equation (4) will always result in estimates that are too low. By the same token, using Cronbach’s alpha values of both components in the congeneric model to “double-correct” the correlation between the manifest composite scores as shown in Equation (5) results in an overestimation of the latent correlation. Thus, using the “double correction” in the congeneric model should produce a number of type 1 errors, i.e. falsely detecting a lack of distinctiveness between the two components. The double correction can therefore be seen as an “upper bound” of distinctiveness. As a consequence, we suggest an additional estimation which corrects for only the lower reliability (i.e. for the component with the broader bandwidth; “single correction”). This “single correction” procedure with only the lower Cronbach’s alpha value (α_*min*_) will underestimate the true latent correlation between *X* and *Y* (e.g. Hakstian et al., 1989) and thus result in a certain probability of type 2 errors, i.e. falsely assuming distinctiveness when indeed the Fornell–Larcker criterion is violated. Thus, a “lower bound” of distinctiveness can be approximated using the manifest Fornell–Larcker criterion with single correction.

$A\,V\,E_{X}>\varphi_{x\,y}^{2}\,\mathrm{which}\,\mathrm{is}\,\mathrm{approximated}\,\mathrm{by}$

$$\frac{\alpha_{x}}{\alpha_{x}*\left(-K_{x}\right)+\alpha_{x}+K_{x}}>\frac{r_{x\,y}}{\sqrt{\alpha_{\min}}}\,\text{and}$$

$A\,V\,E_{y}>\varphi_{x\,y}^{2}\,\mathrm{which}\,\mathrm{is}\,\mathrm{approximated}\,\mathrm{by}$

(7) $$\frac{\alpha_{y}}{\alpha_{y}*\left(-K_{y}\right)+\alpha_{y}+K_{y}}>\frac{r_{x\,y}}{\sqrt{\alpha_{\min}}}$$

The original Fornell–Larcker criterion and therewith a “true” measure of distinctiveness should always be between the manifest Fornell–Larcker criterion with single correction (Equation 7) and with double correction (Equation 6), respectively. Therefore, if both the single and the double correction criterion, i.e. the “lower bound” and “upper bound” of distinctiveness point into the same direction, one can be sure that the result is correct. In the following, we will abbreviate the original “true” Fornell–Larcker criterion as *oFL* and the manifest Fornell–Larcker criterion, which is estimated using Cronbach’s alpha and the correlation matrix between the manifest composite scores, as *mFL.*

## Testing the Manifest Fornell–Larcker Criterion by Means of a Computational Simulation

The goal of this simulation study is to test whether *mFL* can be used to discern lack of distinctiveness in multidimensional instruments. More specifically, the simulation study tests whether *mFL* with “double correction” and “single correction” represents meaningful upper and lower bounds for *oFL* and can therefore be used as an appropriate substitute.

The design of the Monte Carlo simulation follows the one described in Henseler et al. 2015, p. 123): Its population model builds on a two-variable-model with three indicators each. Similar to that of Henseler et al. (2015), we vary the indicator loading patterns to allow for varying degrees of heterogeneity between the loadings, resulting in one essentially tau-equivalent model and five congeneric models. Specifically, we consider the following six loading patterns:

1. λ_*x*.1_ = λ_*y*.1_ = λ_*x*.2_ = λ_*y*.2_ = λ_*x*.3_ = λ_*y*.3_ = 0:70
2. λ_*x*.1_ = λ_*y*.1_ = 0.65; λ_*x*.2_ = λ_*y*.2_ = 0.70; λ_*x*.3_ = λ_*y*.3_ = 0.75
3. λ_*x*.1_ = λ_*y*.1_ = 0.60; λ_*x*.2_ = λ_*y*.2_ = 0.70; λ_*x*.3_ = λ_*y*.3_ = 0.80
4. λ_*x*.1_ = λ_*y*.1_ = 0.55; λ_*x*.2_ = λ_*y*.2_ = 0.70; λ_*x*.3_ = λ_*y*.3_ = 0.85
5. λ_*x*.1_ = λ_*y*.1_ = 0.50; λ_*x*.2_ = λ_*y*.2_ = 0.70; λ_*x*.3_ = λ_*y*.3_ = 0.90
6. λ_*x*.1_ = λ_*y*.1_ = 0.45; λ_*x*.2_ = λ_*y*.2_ = 0.70; λ_*x*.3_ = λ_*y*.3_ = 0.95

Moreover, we vary the inter-variable correlation φ_*xy*_ in 51 steps of 0.02 from φ_xy_ = 1.0 to φ_xy_ = 0.0. Finally, we consider two different sample sizes of 250 and 1,000, respectively. For each of the 612 combinations of design factors, we generated 1,000 datasets, resulting in 612,000 simulation runs in total. In each simulation run, we assessed the following information:

1. *oFL* calculated using *AVE*_x_, *AVE*_y_, and φ_xy_: The other criteria proposed here are compared to this “gold standard.”
2. *mFL* calculated with “double correction”: In the essentially tau-equivalent case, this should be identical to *oFL*, whereas it should produce a number of type 1 errors (falsely detecting a lack of distinctiveness) in the congeneric model.
3. *mFL* calculated with “single-correction”: Compared to *oFL*, this should result in a number of type 2 errors (falsely assuming distinctiveness when indeed *oFL* is violated).

**FIGURE 1:**
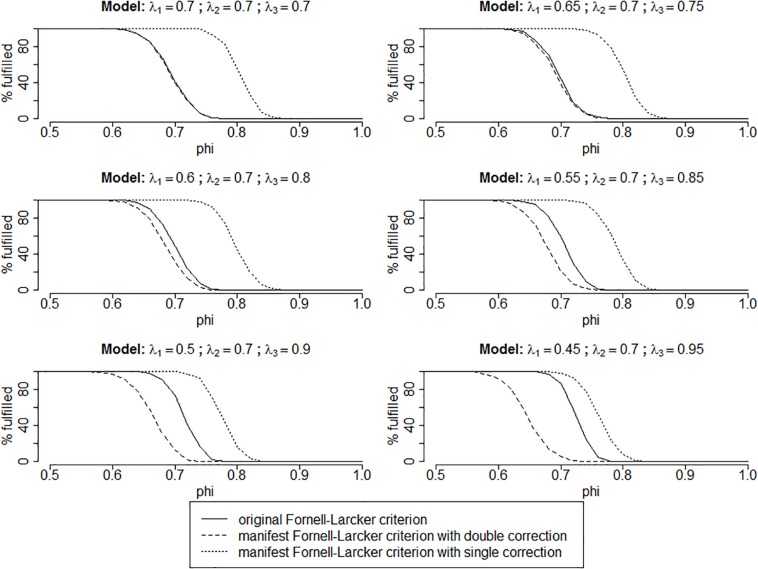
Results of the simulation study for cases with *n* = 1,000. The results of the simulation runs with *n* = 250 are practically identical and therefore not shown here. Moreover, cases with *φ*_*xy*_ < 0.5 are not shown since there are no violations of distinctiveness with the lambdas utilized in this simulation and none of the criteria proposed “detects” any violations either.

All calculations were carried out with R (R Core Team, 2017), using the packages lavaan (Rosseel, 2012), psych (Revelle, 2019), and semTools (Jorgensen et al., 2019); the results are depicted in Figure 1. The graphs visualize the percentage with which each criterion indicates that distinctiveness is met for varying levels of intercorrelations and loading patterns.

As can be seen in the upper left panel of Figure 1, all results are as expected: In the essentially tau-equivalent case, *mFL* with double correction is identical to *oFL*.

The remaining panels of Figure 1 show the congeneric cases. Here, *oFL* is always located between *mFL* with double correction and *mFL* with single correction, supporting that *mFL* is indeed an appropriate substitute. Moreover, as anticipated in the congeneric cases, *mFL* with double correction “detects” violations of distinctiveness where there are none (the area between the dashed line and the solid line); however, *mFL* with single correction misses a number of violations (the area between the solid line and the dotted line). Whether there are more type 1 or type 2 errors depends on the deviation of the data from essential tau-equivalence, with more type 1 and less type 2 errors for stronger deviations from essential tau-equivalence.

The results of the simulation study show that *mFL* can indeed be used to discern lack of distinctiveness in multidimensional instruments. If researchers or practitioners want to be sure that there are no violations of distinctiveness between dimensions, they should use *mFL* with double correction. If this criterion does not detect any violations, there are none. Likewise, when they use *mFL* with single correction and do detect violations, they can be sure that these are indeed correct. We suggest estimating both criteria. When both point in the same direction, one can be sure that the result is correct. Otherwise, it is uncertain if there is any violation of distinctiveness.

## Testing the Usefulness of the Manifest Fornell–Larcker Criterion by Means of a Systematic Review

As shown by Crutzen and Peters (2017), only a fraction of multidimensional instruments explicitly report validity evidence regarding their internal structure, and those articles do not seldom report all necessary information to calculate *oFL a posteriori* (Schmitt and Sass, 2011). The current systematic review shall therefore demonstrate the applicability, utility, and necessity of the proposed *mFL*.

Applicability means that, since *mFL* is estimated with information that are more commonly reported than the information necessary to calculate *oFL*, it should be applicable to a number of studies for which otherwise no validity evidence regarding the internal structure could be estimated.

Utility means that *mFL* should be agnostic to the particular structure of the multidimensional instrument, i.e. it should not favor multidimensional instruments with few components or few items. Otherwise, its real-world usage would be limited.

Necessity means that, based on the results of Cizek et al. (2008), Cook et al. (2014), or Crutzen and Peters (2017), we would expect that the current systematic review will unearth a number of multidimensional instruments that indeed lack distinctiveness between their components, confirming the need for an *auxiliary tool* like *mFL*.

The systematic review used the 2015 volumes of “Frontiers in Psychology,” “Journal of Personality Assessment,” and “Psychological Assessment” searching for “scale,” “measure,” “instrument,” “inventory,” and “questionnaire.” “Frontiers in Psychology” was chosen since it is the largest and second-most cited journal in psychology (Thomson Reuters, 2016; impact factor of 2.463). Moreover, it is an open-access journal, meaning that if a practitioner is interested in a measurement instrument for a certain topic, it is quite likely that she will end up with an instrument published in “Frontiers” simply because the instrument is not behind a paywall and therefore practitioners have unrestricted access to it [see Gargouri et al. (2010) for related findings]. The “Journal of Personality Assessment” (impact factor of 2.258; Thomson Reuters, 2016) was chosen since it is the official journal of the Society for Personality Assessment, the largest psychological society worldwide focused on personality assessment. “Psychological Assessment” (impact factor of 2.901; Thomson Reuters, 2016) was chosen as “the premier assessment journal for APA, [which] should be an exemplar of good psychometric reporting practice for all APA journals in which psychological measures are used” (Green et al., 2011, p. 657). We think that this selection of journals reflects the heterogeneity of psychological journals quite well, with one looking back on a more than 80-year-long tradition and one established as recent as 2010, while all of them being within the top 30% of psychological journals when it comes to the 2 and 5 years impact factor (Thomson Reuters, 2016).

As shown in Table 1, our search yielded 151 unique results, 71 of which dealt with multidimensional instruments. However, only 10 of these 71 articles report any test on distinctiveness well in line with the results of Crutzen and Peters (2017). Of the 71 journal articles, 12 provided all necessary information to compute *oFL* (i.e. factor loadings and latent correlations), and another 29 provided all necessary information to estimate *mFL*. This shows that *mFL* can be applied to a number of studies for which otherwise no validity evidence regarding the internal structure could be estimated.

**TABLE 1:** Unique results of the literature research.

	Unique results	Multi-dimensional instruments	Distinctive- ness mentioned	Information available	mFL met
Frontiers in Psychology	41	19	4	9	5
Journal of Personality Assessment	28	16	3	9	4
Psychological Assessment	79	36	3	23	8

*Search was performed for all volumes of 2015 with the search terms “scale”, “measure”, “instrument”, “inventory”, and “questionnaire.” Information available = all necessary information to compute or estimate the (manifest) Fornell–Larcker criterion are available in the article. mFL, met = manifest Fornell–Larcker criterion is met. The calculations with single correction and double correction led to exactly the same results, suggesting that the difference between the two estimations is actually quite small. When all information to compute the latent Fornell–Larcker criterion were provided, the latent criterion was computed instead.*

As can also be seen in Table 1, nearly 60% of all examined multidimensional scales lack distinctiveness between their respective components. This percentage did not significantly differ between the three journals (χ^2^ [2, *N* = 41] = 1.19, *p* = 0.55). Importantly, these violations are not isolated incidents either. On average, if at least one pair of dimensions lacks distinctiveness, 56% of all non-redundant correlations violate *mFL*, suggesting a severe violation at large and emphasizing the necessity for such an auxiliary tool.

Importantly, our systematic review also shows that the number of non-redundant correlations between the dimensions (and therewith the number of potential violations) of a given instrument is not significantly related to whether this instrument does meet *mFL* or not (*t*[39] = 1.52, *p* = 0.137). Likewise, the average number of items in a given dimension is not significantly related to whether this instrument does meet *mFL* or not (*t*[39] = 0.12, *p* = 0.906), suggesting that the proposed method is indeed agnostic to the particular structure of the multidimensional instrument (i.e. it does not favor multidimensional instruments with few components and few items each), thus indicating its utility.

## General Discussion

The systematic review illustrates that, even with peer-reviewed studies in well-received journals, one should pay close attention to whether the instrument in question validly measures what it is purported to measure. Estimating *mFL* is a useful and efficient way in helping to answer this question. More often than not, researchers and practitioners will find the necessary information within the journal article to *a priori* assess validity evidence based on the internal structure, specifically of the distinctiveness, by themselves. Alternatively, one can use the accompanying website https://hibobohi.github.io/, which computes both *mFL* with double correction and *mFL* with single correction. Again, we suggest estimating both criteria. When both point into the same direction (as they did for every instrument investigated in our systematic review), one can be sure that the result is correct. Only when the double correction does indicate violations and the single correction does not is it uncertain if there is any violation of distinctiveness.

However, the systematic review also shows that about 42% of the multidimensional instruments under investigation do not report sufficient information to calculate the *mFL* criterion. The actual choice on how to proceed then depends on the researcher’s assessment and the emphasis she wants to place on interpreting dimensions separately. We would suggest to (a) contact the authors of the original study and request the raw data to conduct the required analyses on one’s own. After all, if the study is published in an APA journal, the authors had to sign the “APA Certification of Compliance with APA Ethical Principles”, which includes the principle on sharing data for reanalysis (statement 8.14). However, since Wicherts et al. (2006) report that chances of receiving raw data that way are actually quite slim, we would suggest to (b) continue searching for an adequate substitute, i.e. another measurement instrument aimed at the same construct, but with possibly sufficient information.

Thus, *mFL* is no silver bullet. It can improve the decision-making process of researchers and practitioners interested in a certain multidimensional instrument, but only if some basic information is available. Therefore, and perhaps somewhat unconventional, we hope that the necessity of our method vanishes over time: Calculating *mFL* is an *auxiliary tool* to assess the internal structure of a multidimensional measurement instrument, something that could—and should—easily be provided by the authors introducing or using an instrument in the first place. Following Crutzen and Peters, 2017, p. 246), we reemphasize their recommendation that authors include—and editors and reviewers demand—information about the validity of their operationalizations that go beyond Cronbach’s alpha. “The tools to do so are available […], it is up to all of us to take this step towards more insight into scale quality.” However, until the publishing behavior actually changes [and see, e.g. McNeish (2018) for a rather grim outlook on that], *mFL* offers researchers and practitioners an easily assessable method for vetting existing measurement instruments and thus helps them choose better “common tools” (Mischel, 2009) to obtain more robust, replicable, and consequential findings on our way to a more integrative science.

## Data Availability Statement

This study re-analyzed the information provided in the research articles of the 2015 volumes of “Frontiers in Psychology,” “Journal of Personality Assessment,” and “Psychological Assessment”. The information can be found in the respective articles.

## Author Contributions

FH, CB, TB, and JH all contributed to the conception of the overall method. TB and CB designed and computed the simulation study. FH, CB, and JH designed and carried out the systematic review. FH performed the statistical analysis and wrote the first draft of the manuscript. CB wrote sections of the manuscript. All authors contributed to manuscript revision, read and approved the submitted version.

## Conflict of Interest

The authors declare that the research was conducted in the absence of any commercial or financial relationships that could be construed as a potential conflict of interest.

## Footnotes

1 Distinctiveness between components is commonly also referred to as “discriminant validity.” However, as initiated by Messick (1995) and reflected in the standards (American Educational Research Association [AERA] et al., 2014), the view and terminology of validity recently shifted to a more unitary perspective. In this more recent terminology, “discriminant evidence for validity” is exclusively used for external aspects of construct validity, referring to the relationship of the focal instrument to other measures. Discrimination of subdimensions now falls under “evidence based on internal structure” (American Educational Research Association [AERA] et al., 2014). In order to adhere to the terminology of the standards, we here embrace the term “distinctiveness” instead of “discriminant validity.”
