# Supplementary material for: Assessing Distinctiveness in Multidimensional Instruments Without Access to Raw Data – A Manifest Fornell-Larcker Criterion
Source: Front Psychol. 2020 Mar 9;11:223. doi: 10.3389/fpsyg.2020.00223 (PMC7075488; doi:10.3389/fpsyg.2020.00223)
Supplement: Supplementary file 1 [file Data_Sheet_1.pdf]

Assessing Distinctiveness in Multidimensional Instruments without Access to Raw Data – A Manifest Fornell-Larcker Criterion

Frederic Hilkenmeier, Carla Bohndick, Thomas Bohndick and Johanna Hilkenmeier

Frontiers in Psychology, 11:223. doi: 10.3389/fpsyg.2020.00223

Electronic Supplementary Material

Electronic Supplementary Material 1:

Detailed derivation of the equations used to substitute the AVE and the correlation between the latent variables in the essentially tau-equivalent Model

Note: The numbering of the equations, tables and figures corresponds to the numbering in the article.

The original Fornell-Larcker criterion

The formal definition of the *AVE* of a given latent variable X with standardized indicators can be seen in Equation (1).

$$AVE_x = \frac{\sum \lambda^2{}_{x.i}}{K_x} \quad (1)$$

where $\lambda^2{}_{x.i}$ is the squared loading of indicator $x.i$ on the latent variable X and K_x is the number of indicators associated with X. As seen in Equation (2), the Fornell-Larcker criterion and thus the requirements for distinctiveness between two latent variables X and Y are fully met if the *AVE* of X and Y are both higher than the variance X and Y share with each other.

$$AVE_X > \varphi^2_{xy} \text{ and } AVE_y > \varphi^2_{xy} \tag{2}$$

where φ^2_{xy} is the squared correlation between X and Y. Figure 0a gives an example of two latent variables with three manifest indicators each.

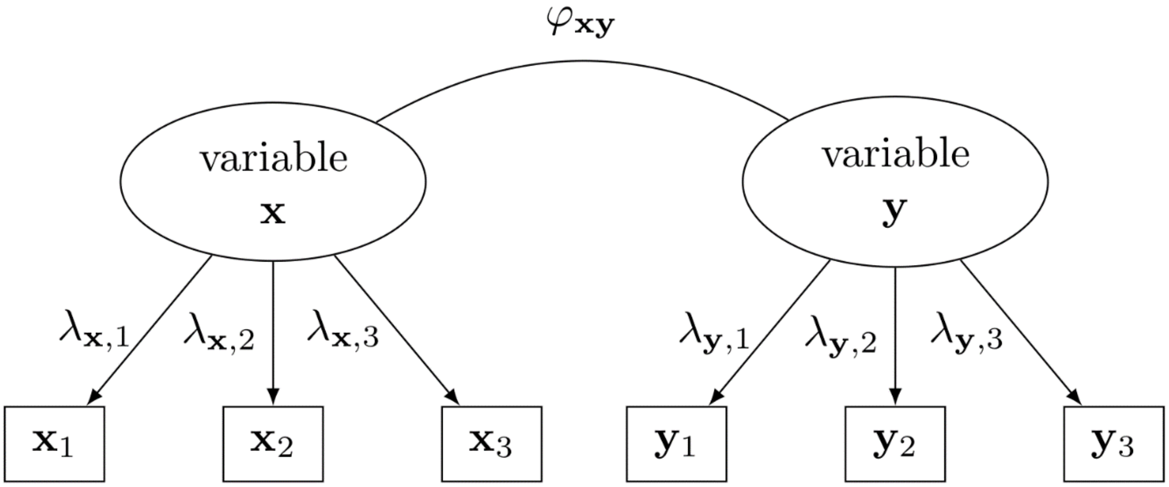

$$\mathbf{AVE_x} = \frac{\lambda^2_{\mathbf{x},1}+\lambda^2_{\mathbf{x},2}+\lambda^2_{\mathbf{x},3}}{3} \qquad \mathbf{AVE_y} = \frac{\lambda^2_{\mathbf{y},1}+\lambda^2_{\mathbf{y},2}+\lambda^2_{\mathbf{y},3}}{3}$$

*Figure 0a***.** Example of two latent variables with three manifest indicators each.

Substitute for the AVE in the Essentially Tau-Equivalent Model

According to Sijtsma (2009, p.107, also see McNeish, 2018), "probably no other statistic has been reported more often as a quality indicator of test scores than Cronbach's (1951) alpha coefficient". If the items of an instrument are essentially tau-equivalent, Cronbach's alpha is true indicator of that instrument's reliability (e.g., Graham, 2006; Miller, 1995). The formula for standardized Cronbach's alpha (which in the essentially tau-

equivalent model is identical to Cronbach’s alpha, but easier to calculate and, more importantly, based on the correlation instead of the covariance) is as follows:

$$\alpha_x = \frac{\sum r_i}{1 + (K_x - 1) * (\frac{\sum r_i}{K_x})} \quad (3)$$

where α_x is the standardized Cronbach’s alpha of all indicators associated with the latent variable X and r_i is the interitem correlation of indicator i with all other indicators associated with X. What is clear from Equation (3) is that standardized Cronbach’s alpha is simply a function of the average interitem correlation of all indicators associated with a given variable and the number of indicators. It is by no means a test for the internal structure. In fact, the assumption of essential tau-equivalence requires that the measurement instrument is homogeneous and unidimensional. Such an essentially tau-equivalent model is shown in the left part of Table 0. We will use this example throughout the electronic supplementary material. As we will discuss subsequently, in the more realistic congeneric model Cronbach’s alpha generally underestimates the reliability of the measurement instrument. Alternatives that are more appropriate are readily available. However, as shown by Hogan, Benjamin, and Brezinski (2000), and most recently McNeish (2018), none of these more appropriate alternatives are actually reported, the overwhelming majority of studies only reports Cronbach’s alpha. Since the manifest Fornell-Larcker criterion is specifically designed to estimate distinctiveness based on information most likely reported in a given study, all of the calculations that follow are based on Cronbach’s alpha as well.

Table 0

Intercorrelation matrices for two sets of items with identical Cronbach's alpha

	x_1	x_2	x_3		y_1	y_2	y_3
x_1	-			y_1	-		
x_2	.3	-		y_2	.7	-	
x_3	.3	.3	-	y_3	.1	.1	-
average interitem correlation $r_i = .3$				average interitem correlation $r_i = .3$			
Cronbach's alpha = .56				Cronbach's alpha = .56			

Note. This table is inspired from Cortina, 1993, p. 100.

In the essentially tau-equivalent model, the average loading of all indicators associated with a variable $\frac{\sum \lambda_{x.i}}{K_x}$ is equivalent to the square root of the average manifest interitem correlation of these indicators $\sqrt{\frac{\sum r_i}{K_x}}$ (John & Benet-Martinez, 2000, p.345; John & Soto, 2009, p.486).

$$\frac{\sum \lambda_{x.i}}{K_x} = \sqrt{\frac{\sum r_i}{K_x}} \tag{3b}$$

Moreover, given essential tau-equivalence, all $\lambda_{x.i}$ are equal, thus, the *squared average* loading $\left(\frac{\sum \lambda_{x.i}}{K_x}\right)^2$ is identical to the *average squared* loading $\frac{\sum \lambda^2{}_{x.i}}{K_x}$.

$$\left(\frac{\sum \lambda_{x.i}}{K_x}\right)^2 = \frac{\sum \lambda^2{}_{x.i}}{K_x} \tag{3c}$$

As shown in Equation (3) standardized Cronbach's alpha is a function of the average interitem correlation of all indicators and the number of indicators. Equation (3) may thus be rearranged to give

$$\frac{\sum r_i}{K_x} = \frac{\alpha_x}{\alpha_x * (-K_x) + \alpha_x + K_x} \tag{3d}$$

Thus, the standardized Cronbach's alpha and the number of items are sufficient to calculate the AVE.

$$AVE_x = \frac{\sum \lambda^2{}_{x.i}}{K_x} = \left(\frac{\sum \lambda_{x.i}}{K_x}\right)^2 = \frac{\sum r_i}{K_x} = \frac{\alpha_x}{\alpha_x * (-K_x) + \alpha_x + K_x} \tag{4}$$

Substitute for the Correlation between the Latent Variables in the Essentially Tau-Equivalent Model

Again, the Fornell-Larcker criterion was developed to assess distinctiveness between latent variables. However, many papers only report the correlation matrix between the composite scores (i.e. the summed scores or mean scores), not between the latent ones. Unlike correlations between latent variables, correlations between manifest variables do not take measurement error into account. This measurement error in manifest variables is likely to mask the true correlation between the variables, resulting in an attenuated correlation (e.g., Block, 1963). This means correlations between manifest variables are generally lower than correlations between their latent counterparts (Farrell, 2010; Grewal, Cote, & Baumgartner, 2004; Lord & Novick, 1974, p. 68; Nunnally, 1978, p. 237). To account for the measurement error in manifest correlations, one can "correct" for this attenuation as shown in Equation(4b)

$$\varphi_{xy} = \widehat{r_{xy}} = \frac{r_{xy}}{\sqrt{r_{xx}} * \sqrt{r_{yy}}} \tag{4b}$$

where $\widehat{r_{xy}}$ is the "corrected" ("double corrected", to be more precise) correlation between the two composite scores of X and Y, r_{xy} is the manifest correlation between the two composite scores of X and Y, and r_{xx} and r_{yy} are the reliability of X and Y, respectively (e.g., Bedeian, Day, & Kelloway, 1997; Nunnally, 1978, p. 219; also see Spearman, 1904). As discussed previously, in the essentially tau-equivalent model, Cronbach's alpha is a true measure of reliability (e.g., Novick & Lewis, 1967, Theorem 3.1; Raykov, 1997; Sijtsma, 2009, p.111). Therefore,

$$\varphi_{xy} = \widehat{r_{xy}} = \frac{r_{xy}}{\sqrt{\propto_x} * \sqrt{\propto_y}} \quad (5)$$

This shows that in the essentially tau-equivalent model, distinctiveness (as understood by Fornell and Larcker) can indeed be calculated using nothing but Cronbach's alpha and the manifest correlation between the composite scores.

$AVE_X > \varphi^2{}_{xy}$ which is equivalent to $\frac{\alpha_x}{\alpha_x * (-K_x) + \alpha_x + K_x} > \frac{r_{xy}}{\sqrt{\alpha_x} * \sqrt{\alpha_y}}$ and

$AVE_y > \varphi^2{}_{xy}$ which is equivalent to $\frac{\alpha_y}{\alpha_y * (-K_y) + \alpha_y + K_y} > \frac{r_{xy}}{\sqrt{\alpha_x} * \sqrt{\alpha_y}}$ (6)

Electronic Supplementary Material 2:

Detailed derivation of the equations used to substitute the AVE and the correlation between the latent variables in the congeneric Model

Substitute for the AVE in the Congeneric Model

Now we will turn to the congeneric model, which is much more realistic for empirical data (e.g. Graham, 2006; Miller, 1995), but at the same time fuzzier when it comes to deriving the equations. When the assumption of essential tau-equivalence is violated, Cronbach's alpha will underestimate the reliability of the measurement instrument and therewith its AVE (Raykov, 1997). Consider again the example given in Table 0: On the left part the data are essentially tau-equivalent. Thus, following Equation (3b), the loadings $\lambda_{x.1}$ – $\lambda_{x.3}$ are all .55 and therefore, as shown in Equation (1), AVE_x is .30. However, for the right part of Table 1, the associated loadings $\lambda_{y.1}$ – $\lambda_{y.3}$ are .84; .84; .12, with AVE_y resulting in .48, whereas, based on Cronbach's alpha and given Equation (4), we would again estimate the AVE_y to be .30.

As can be seen in Figure 0b, while the Cronbach's alpha coefficient stays the same, the AVE increases as a function of the variability of these loadings (and thus the variability of the underlying intercorrelation matrix; see Raykov, 1997 for an in-depth analysis of the conditions under which Cronbach' alpha underestimates reliability).

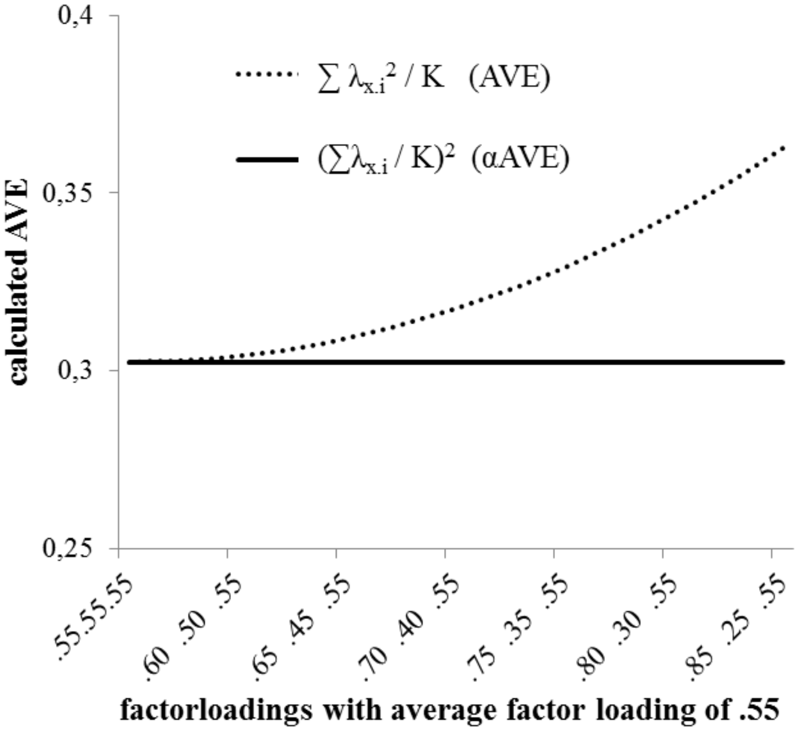

Figure 0b. Calculated *AVE* as a function of the variability of loadings for a variable with 3 manifest indicators. The dotted line represents the correct calculation of the *AVE* as shown in Equation (1), whereas the solid line represents the *AVE* calculated using Cronbach's alpha as shown in Equation (4) and only viable in cases of essential tau-equivalence.

Therefore, we cannot assume Equations (3b), (3c), and (4) to be true for empirical data, which seldom meet the criteria of the essentially tau-equivalent model.

In the congeneric model, $\left(\frac{\sum \lambda_{x.i}}{K_x}\right)^2$ approximates $\frac{\sum \lambda^2{}_{x.i}}{K_x}$ while always resulting in lower estimates, changing Equation (3c) to

$$\left(\frac{\sum \lambda_{x.i}}{K_x}\right)^2 < \frac{\sum \lambda^2{}_{x.i}}{K_x} \quad \text{(6b)}$$

Moreover, when the data are not essentially tau-equivalent, the square root of the average manifest interitem correlation of the indicators associated with a given variable is *not* equivalent to the average loading of all indicators associated with that variable. For the right part of Table 1, the average loading of the indicators $\lambda_{y.1} - \lambda_{y.3}$ is .60, whereas the square root of the average manifest interitem correlation of the indicators $_{y.1}$ - $_{y.3}$ is .55, changing Equation (3b) to

$$\frac{\sum \lambda_{x.i}}{K_x} \cong \sqrt{\frac{\sum r_i}{K_x}}, \qquad (6c)$$

with stronger deviations for stronger violations of essential tau-equivalence. However, even though $\sqrt{\frac{\sum r_i}{K_x}}$ might vary from $\frac{\sum \lambda_{x.i}}{K_x}$, $\frac{\sum r_i}{K_x}$ is always lower than $\frac{\sum \lambda^2{}_{x.i}}{K_x}$ (Raykov, 1997), resulting in Inequality (6d).

$$\frac{\sum \lambda^2{}_{x.i}}{K_x} > \frac{\sum r_i}{K_x} \qquad (6d)$$

Given Equation (3d), the *AVE* can be approximated (while always resulting in lower estimates) using nothing but the standardized Cronbach's alpha and the number of items

$$AVE_x = \frac{\sum \lambda^2{}_{x.i}}{K_x} > \frac{\sum r_i}{K_x} = \frac{\alpha_x}{\alpha_x * (-K_x) + \alpha_x + K_x} \qquad (6e)$$

Since we usually do not know whether data are essentially tau-equivalent or not, or to what degree they violate the assumption of essential tau-equivalence, especially when we have no access to raw data, we generalize inequality (6e) to

$$AVE_x > \alpha AVE_x \qquad (6f)$$

where αAVE_x is the average variance extracted of a given X using nothing but the standardized Cronbach's alpha coefficient and the number of items associated with the given X and $AVE_x = \alpha AVE_x$ holds if and only if all indicators are essentially tau-equivalent. However, according to Raykov (1997, p. 347, also see Raykov & Marcoulides, 2017), Cronbach's alpha still "represents a "good" measure of composite reliability [*AVE*] at the population level, in particular with other than short scales."

Substitute for the Correlation between the Latent Variables in the Congeneric Model

Again, in the congeneric model, Cronbach's alpha somewhat underestimates the reliability of a given instrument, resulting in an underestimation of its *αAVE*. By the same token, using Cronbach's alpha in the congeneric model to "double correct" the correlation between the manifest composite scores as shown in Equation (4b) would result in an overestimation of the latent correlation (e.g., Muchinsky, 1996), changing Equation (5) to

$$\varphi_{xy} < \frac{r_{xy}}{\sqrt{\alpha_x} * \sqrt{\alpha_y}} \tag{6g}$$

Using Inequality (6g) to assess distinctiveness thus results in a higher probability of type 1 errors, meaning the probability that one "detects" a violation of the Fornell-Larcker criterion (and thus a lack of distinctiveness) where there is none increases. Not using any correction however, leads to a high probability of type 2 errors, meaning one falsely assumes distinctiveness when indeed the Fornell-Larcker criterion is violated (see Block, 1963 for a related argument). As a consequence, we suggest *also* correcting for only the lower reliability (i.e. for the component with the broader bandwidth; "single correction"). This "single correction" procedure with only the lower Cronbach's alpha value will still underestimate the true latent correlation between X and Y and thus there is still a certain probability of type 2

errors, but a much lower one then without any correction (e.g. Hakstian, Schroeder, & Rogers, 1989).

$$\varphi_{xy} > \frac{r_{xy}}{\sqrt{\alpha_{\min}}} \qquad (6h)$$

where $\alpha_{\min}$ is the lower reliability of the two components X and Y. Taken together, in the congeneric model, distinctiveness between two variables can be approximated by using Cronbach's alpha and the manifest correlation between the composite scores.

$$AVE_X > \varphi^2{}_{xy} \text{ which is approximated by } \frac{\alpha_x}{\alpha_x * (-K_x) + \alpha_x + K_x} > \frac{r_{xy}}{\sqrt{\alpha_{\min}}} \text{ and}$$

$$AVE_y > \varphi^2{}_{xy} \text{ which is approximated by } \frac{\alpha_y}{\alpha_y * (-K_y) + \alpha_y + K_y} > \frac{r_{xy}}{\sqrt{\alpha_{\min}}} \qquad (7)$$

References

Bedeian, A. G., Day, D. V., & Kelloway, E. K. (1997). Correcting for measurement error attenuation in structural equation models: Some important reminders. *Educational and Psychological Measurement, 57(5),* 785-799. doi: 10.1177/0013164497057005004

Block, J. (1963). The equivalence of measures and the correction for attenuation. *Psychological Bulletin, 60,* 152-156. doi: 10.1037/h0041811

Cronbach, L. J. (1951). Coefficient alpha and the internal structure of tests. *Psychometrika, 16,* 297-334.

Farrell, A. M. (2010). Insufficient discriminant validity: a comment on Bove, Pervan, Beatty, and Shiu (2009). *Journal of Business Research, 63,* 324-327. doi: 10.1016/j.jbusres.2009.05.003

Fornell, C., & Larcker, D. F. (1981). Evaluating structural equation models with unobservable variables and measurement error. *Journal of Marketing Research, 18,* 39-50. doi: 10.2307/3151312

Graham, J. M. (2006). Congeneric and (essentially) tau-equivalent estimates of score reliability – What they are and how to use them. *Educational and Psychological Measurement, 66 (6),* 930-944. doi: 10.1177/0013164406288165

Grewal R., Cote, J. A., & Baumgartner, H. (2004). Multicollinearity and measurement error in structural equation models: implications for theory testing. *Marketing Science, 23(4),* 519-29. doi: 10.1287/mksc.1040.0070

Hakstian, A. R., Schroeder, M. L., & Rogers, W. T. (1989). Inferential theory for partially disattenuated correlation coefficients. *Psychometrika, 54,* 397-407. doi: 10.1007/BF02294625

Hogan, T. P., Benjamin, A., & Brezinski, K. L. (2000). Reliability methods: A note on the frequency of use of various types. *Educational and Psychological Measurement, 60,* 523-531. doi: 10.1177/00131640021970691

John, O. P., & Benet-Martínez, V. (2000). Measurement, scale construction, and reliability. In H.T. Reis and C.M. Judd (Eds.), *Handbook of research methods in social and personality psychology* (pp.339-369). New York: Cambridge University Press.

John, O. P., & Soto, C. J. (2007). The importance of being valid: Reliability and the process of construct validation. In Robins, R. W., Fraley, R. C., & Krueger, R. F. (Eds.), *Handbook of research methods in personality psychology* (pp. 461-494). New York: Guilford.

Lord, F. M., & Novick, M. R. (1974). *Statistical theories of mental test scores. Reading. MA: Addison Wesley.*

McNeish, D. (2018). T Thanks coefficient alpha, we'll take it from here. *Psychological Methods, 23,* 412 - 433. doi: 10.1037/met0000144

Miller, M. B. (1995). Coefficient alpha: A basic introduction from the perspectives of classical test theory and structural equation modeling. *Structural Equation Modeling, 2(3),* 255-273. doi: 10.1080/10705519509540013

Muchinsky, P. M. (1996). The correction for attenuation. *Educational and Psychological Measurement, 56(1),* 63-75. doi: 10.1177/0013164496056001004

Novick, M. R., & Lewis, C. (1967). Coefficient alpha and the reliability of composite measurements. *Psychometrika, 32*(1), 1–13. doi:10.1007/BF02289400

Nunnally, J. (1978). *Psychometric theory.* New York: McGraw-Hill.

Raykov, T. (1997). Scale reliability, Cronbach's coefficient alpha, and violations of essential tau equivalence with fixed congeneric components. *Multivariate Behavioral Research, 32,* 329 - 353. doi: 10.1207/s15327906mbr3204_2

Raykov, T., & Marcoulides, G. A. (2017). Thanks coefficient alpha, we still need you! *Educational and Psychological Measurement.* Advance online publication. Doi: 10.1177/0013164417725127

Sijtsma, K. (2009). On the use, the misuse, and the very limited usefulness of Cronbach's alpha. *Psychometrika*, *74(1),* 107-120. doi: 10.1007/s11336-008-9101-0

Spearman, C. (1904). The proof and measurement of association between two things. *American Journal of Psychology*, 15(1), 72-101.
